# Validation of the bowel urgency numeric rating scale in patients with Crohn’s disease: results from a mixed methods study

**DOI:** 10.1007/s11136-023-03494-y

**Published:** 2023-08-04

**Authors:** Marla C. Dubinsky, Laure Delbecque, Theresa Hunter, Gale Harding, Larissa Stassek, Richard E. Moses, James D. Lewis

**Affiliations:** 1 Pediatric GI and Nutrition, Icahn School of Medicine- Mount Sinai Hospital, New York City, NY USA; 2 Eli Lilly and Company, 893 Delaware St, Indianapolis, IN 46225 USA; 3 Evidera, Bethesda, MD USA; 4 Perelman School of Medicine, University of Pennsylvania, Philadelphia, PA USA

**Keywords:** Bowel urgency, Urgency NRS, Numeric rating scale, Crohn's disease, Qualitative interviews

## Abstract

**Purpose:**

Bowel urgency (BU) is an important symptom of Crohn’s disease (CD), however there is no patient-reported outcome (PRO) scale validated in this population to assess BU severity. Here we evaluated the content validity and psychometric properties of the Urgency Numeric Rating Scale (NRS).

**Method:**

Qualitative interviews were conducted with moderate-to-severe CD participants to confirm importance and relevance of BU in this population, cognitively debrief the Urgency NRS, and explore score interpretation and CD remission. A quantitative web survey study was conducted to explore the measurement properties of the urgency NRS.

**Results:**

*Qualitative Interview:* 34 of 35 participants reported BU. It was most bothersome for 44%, 47% reported it daily, 18% with every bowel movement. BU had a severe impact on daily activities, causing many participants to stay home more than preferred. Patients confirmed the relevance, appropriateness, comprehensibility of the item, recall period, response options, and instructions of the Urgency NRS. Small reductions on the Urgency NRS score reflected meaningful improvements. *Quantitative survey:* The study sample comprised 76 participants (65.8% female). Mean Urgency NRS score was 4.7 (SD 2.26; N = 76) at Week 1, with no floor/ceiling effect. Test–retest reliability was acceptable. Construct and known-groups validity against selected PROs were overall strong and within ranges hypothesized a priori.

**Conclusion:**

The Urgency NRS is a valid and reliable instrument to assess BU severity in CD.

**Supplementary Information:**

The online version contains supplementary material available at 10.1007/s11136-023-03494-y.

## Introduction

Crohn’s disease (CD) is a chronic, idiopathic, relapsing to immune mediated disease with recurrent cycles of relapse and remission [1, 2]. Prevalence estimates range between 201 (USA) and 319 (Canada) per 100,000 adults [1, 3]. Incidence was 20.2 per 100,000 person-years in North America, 12.7 per 100,000 person-years in Europe, and 5.0 person-years in Asia and the Middle East [4].

Inflammation associated with CD has transmural character and occur in discontinuous manner along the gastrointestinal (GI) tract.[5]. Bowel urgency (BU), the sudden or immediate need to have a bowel movement, is a frequent [5–7] bothersome [8–12] symptom for CD patients and one of the most important symptoms that patients with CD want to gain control of with treatment [8, 13]. Symptoms associated with CD often correspond to the disease behavior and location [14]. Chronic inflammation in the anorectal region and small intestine, for example, are known to cause symptoms such as BU [5, 15]. However, BU can occur without evidence of bowel inflammation in patients with CD [7, 16].

CD is associated with considerable morbidity and significantly diminished health-related quality of life (HRQOL). New treatments for CD should provide symptomatic relief [17], which is best evaluated with patient reported outcomes (PRO) measures. The US Food and Drug Administration (FDA) is encouraging the development of Patient-Reported Outcome (PRO) measures to be incorporated into clinical testing for new therapies to assess concepts that are important to patients [5, 6]. Clinical trials have historically relied heavily on clinical or endoscopic indices, rather than PRO measures [7]. Consequently, there is a need for the development and validation of PRO instruments that assess important concepts. Currently, there is an absence of PROs that assess BU and that are validated for patients with CD.

The Urgency Numeric Rating Scale (NRS) was developed and validated in ulcerative colitis (UC), and allows respondents to rate the severity of their bowel urgency over the past 24 h using a 0 (“no urgency”) to 10 (“worst possible urgency”) scale [18]. This study was designed to explore the patient experience of CD, confirm importance and relevance of BU in this population, cognitively debrief the Urgency NRS to establish content validity, explore meaningful change and BU remission, and assess its measurement properties.

## Methods

This mixed-method observational study consisted of a cross-sectional qualitative interview component (Part A) and a longitudinal quantitative web-based survey (Part B). Patients were recruited via clinical sites (Part A, with some patients referred also to Part B) and through a research recruitment vendor (Part B only) between August 2020 and February 2021. All participants were adults (18 + years old) with a clinically confirmed diagnosis of CD who lived in the US and spoke English. Clinical diagnosis of CD was confirmed by medical records review for patients referred by clinical sites, or by another accepted form of evidence of diagnosis provided by participants recruited via the research recruitment vendor. Participants provided informed consent prior to taking part in the study. This study was conducted in compliance with Good Clinical Practice guidelines, including International Conference on Harmonization Guidelines. The study protocols were approved by Advarra (Columbia, MD).

### Part A. Qualitative interviews

Six US clinical sites recruited an initial target of 25 adult patients with a documented diagnosis of moderate-to-severe CD, based on clinical, endoscopic, radiologic and laboratory examination per national guidelines for CD diagnosis [19]. Sample size estimation was based on expectations to achieve saturation [20, 21]. A purposive sampling approach was used. All participants were required to be currently experiencing, or have had experienced, CD symptoms within the past three months, based on self-report. Patients were excluded if they had an ileostomy, colostomy, or intra-abdominal surgery within 3 months or had a comorbid condition which may have confounded discussion of their CD symptoms.

Interview recruitment targeted approximately even distribution of patients with each of the following 5 CD subtypes, as reported by the clinical site based on medical records: Type 1: Small bowel involvement only, including isolated ileitis; Type 2: Colonic involvement, with or without small bowel involvement (Proximal ± Transverse Colon only); Type 3: Colonic involvement, with or without small bowel involvement (rectal only); Type 4: Colonic involvement, with or without small bowel involvement (rectal + distal colon only); and Type 5: Colonic involvement, with or without small bowel involvement (pancolitis).

#### Qualitative interview procedures

Telephone interviews conducted by 4 interviewers who were trained in qualitative interview methods. A semi-structured interview guide consisting of concept elicitation and cognitive debriefing sections was used in conducting the interviews. Open-ended questions about participants experiences with CD allowed them to describe their overall symptom experience before focusing on BU. Participants experiencing BU described it in greater detail, its impact on daily life, and if this symptom occurred during CD remission. The interviewer instructed the participants to complete PRO measures of interest (below) and then asked questions about interpretation, clarity, relevance, and feasibility of the individual measures and items. Meaningful change (minimum amount of improvement that would make a treatment worth taking) was discussed for some items. PROs of interest included 3 single items: the Patient Global Impression of Change (PGIC), Urgency NRS and Overall CD Symptom Patient Global Rating of Severity (PGRS) (Supplemental Fig. 1).

**Fig. 1:**
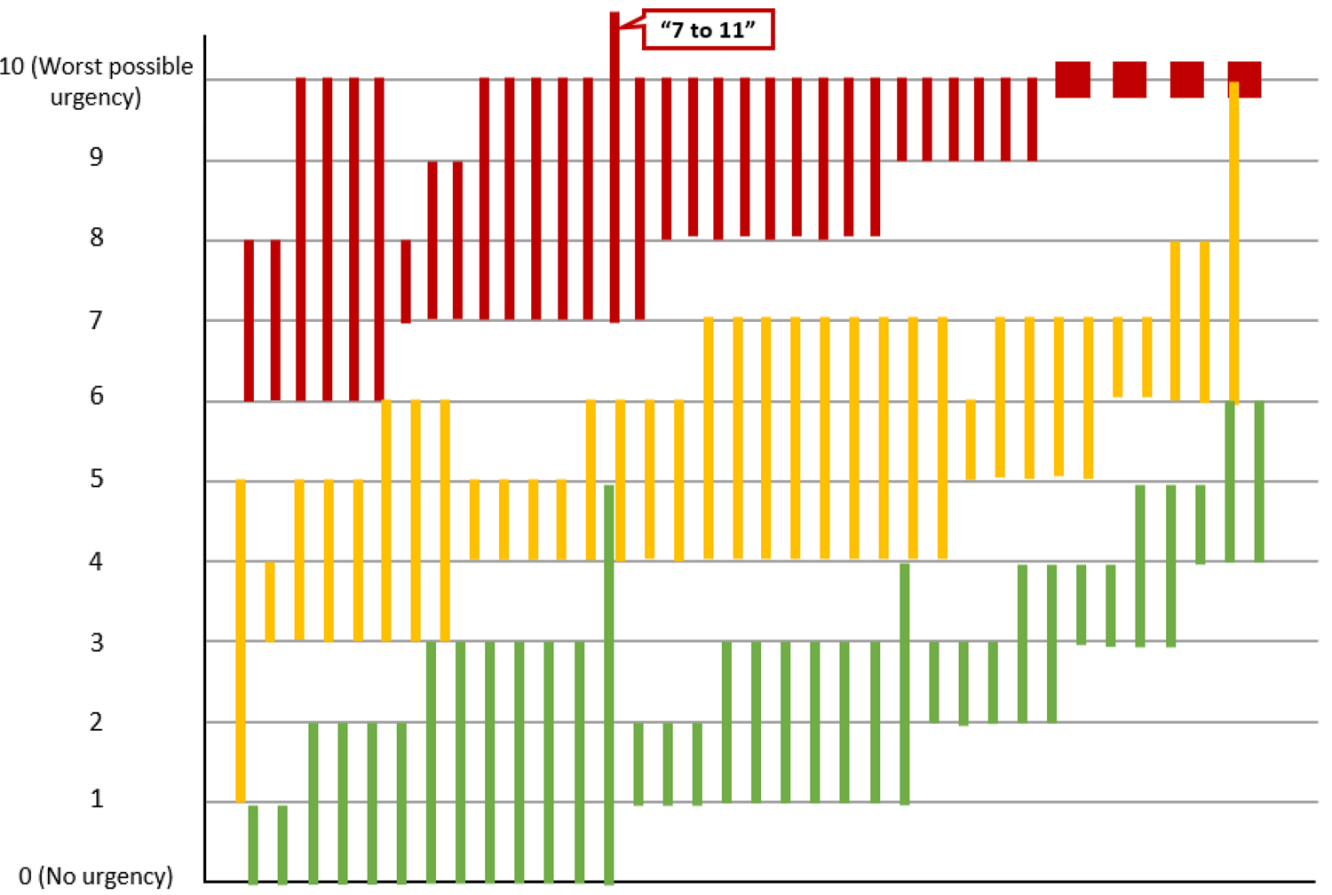
Distribution of Bowel Urgency NRS Severity Level Categories by Individual Participant. Each participant was asked to provide a score range for the 3 levels of severity (ie; mild, moderate, severe); therefore, each participant is represented by 3 bars. Green = “mild” ranges; Yellow = “moderate” ranges; Red = “Severe ranges.” Four participants indicated that “Severe” was “a 10” rather than providing a range; these participants are represented by the red squares

#### Qualitative interview data analysis

Interview transcripts without personal health information were loaded into ATLAS.ti (version 8.0 or higher) for qualitative coding and analysis [22, 23]. A coding dictionary was developed based on the interview guide and the themes and concepts that emerged during the interviews. Coded transcripts were reviewed for quality control. The elicitation data were assessed to document saturation [23, 24] of CD symptom concepts. Interview responses were analyzed to compare and tally the number of novel symptoms that were observed per interview. For the cognitive portion, coding was used to examine the relevance, clarity, and appropriateness of the PRO measures of interest, and to assess participant discussion around meaningful change. Sociodemographic and clinical characteristics were assessed using descriptive statistics.

### Part B. Quantitative web-based survey

The primary web survey recruitment source was the research recruitment vendor, although qualitative interview participants could participate. Participants self-reported a diagnosis of CD that was supported during screening by some form of evidence, such as a signed letter from the participant’s clinician or an official summary of a medical appointment or procedure listing the diagnosis. Participants were excluded if they self-reported having any comorbid health conditions which might have confounded survey responses. Participants reporting CD symptoms in fewer than 10 days of the past month (“asymptomatic”) were allowed to participate, up to 20% of the total survey sample.

#### Web survey procedures

Eligible participants received an email to create an account, provide consent, and start the survey via the vendor’s platform, Baseline Plus, managed by Cisiv. Participants completed survey questions daily for 14 days. Participants were required respond to all questions at all time points; however, an “opt out” response option was provided for participants who did not want to answer a given question. Data was considered “missing” for items where participants used this “opt out” response. Reminders were sent to participants who missed a survey entry day. Participants completing at least the Day 1/Baseline survey entry were remunerated between $75.00 and $225.00 USD based on the number of entries completed.

#### Assessments

The survey administration (Supplemental Table 1) required ≥ 4 responses each week for all measures utilizing a weekly mean score (i.e.; mean of Days 1 through 7 and of Days 8 through 14). Respondents were asked to answer the Urgency NRS during all 14 days of the survey, and two weekly mean scores were calculated. The PROs used to assist in validating the Urgency NRS were overall CD symptoms PGRS (administered daily) and PGIC (administered once), abdominal pain NRS (administered daily), Functional Assessment of Chronic Illness (FACIT)-Fatigue [25] (administered weekly), and Patient Global Impression of Severity (PGIS)-Fatigue [26] (administered weekly). In addition, the Abdominal Pain NRS, an 11-point scale ranging from 0, indicating “no pain” to 10, indicating “Pain as bad as you can imagine,” and the Bowel Movement (BM) Count, that assessed the number of BMs the participant had over the previous 24 h, were also used. The Abdominal Pain NRS and the BM Count were completed daily and mean weekly scores were calculated.

**Table 1:** Patient demographics and disease characteristics

Participant characteristic	Qualitative interview population (N = 35)	Survey population (N = 76)
Age (years)		
Mean (SD)	45.1 (15.83)	41.9 (13.24)
Median [Range]	46.0 [19–74]	40.5 [19–70]
Gender, n (%)		
Male	12 (34.3%)	26 (34.2%)
Female	23 (65.7%)	50 (65.8%)
Ethnicity, n (%)		
Hispanic or Latino	1 (2.9%)	4 (5.3%)
Not Hispanic or Latino	34 (97.1%)	72 (94.7%)
Racial Background^a^, n (%)		
American Indian or Alaska Native	1 (2.9%)	
Asian	1 (2.9%)	2 (2.6%)
Black or African American	3 (8.6%)	11 (14.5%)
Native Hawaiian or Other Pacific Islander	1 (2.9%)	
White	28 (80.0%)	63 (82.9%)
Other: specified “Biracial”	1 (2.9%)	1 (1.3%)
Missing	1 (2.9%)	1 (1.3%)
Employment Status, n (%)		
Employed, full-time	17 (48.6%)	42 (55.3%)
Employed, part-time	5 (14.3%)	10 (13.2%)
Student	3 (8.6%)	5 (6.6%)
Retired	5 (14.3%)	3 (3.9%)
Disabled	4 (11.4%)	9 (11.8%)
Homemaker / Stay-at-home parent		4 (5.3%)
Unemployed		5 (6.6%)
Other	2 (5.7%)^b^	2 (2.6%)^c^
Highest Level of Education, n (%)		
Secondary/high school	11 (31.4%)	4 (5.3%)
Some college	8 (22.9%)	12 (15.8%)
College degree	13 (37.1%)	46 (60.5%)
Postgraduate degree	2 (5.7%)	14 (18.4%)
Other: Specified “massage therapist; STNA [state tested nursing assistant]”	1 (2.9%)	
Marital Status, n (%)		
Single	13 (37.1%)	27 (35.5%)
Married	13 (37.1%)	34 (44.7%)
Divorced	8 (22.9%)	10 (13.2%)
Separated		1 (1.3%)
Widowed	1 (2.9%)	2 (2.6%)
Other		2 (2.6%)^d^
Clinical Characteristic		
Time since diagnosis (years)		
Mean (SD)	11.9 (13.68)	11.8 (11.76)
Median [Range]	6.8 [0–58]	7.5 [0–48]
Most Recent CRP score (mg/L)		
Mean (SD)	19.3 (32.69)	
Median [Range]	7.1 [0–132]	
Unavailable	13 (37.1%)	
Current Treatment^a^, n (%)		
Biologics		56 (73.7%)
Adalimumab	7 (20.0%)	
Certolizumab	3 (8.6%)	
Infliximab	3 (8.6%)	
Ustekinumab	6 (17.1%)	
Tofacitinib	1 (2.9%)	
Vedolizumab	4 (11.4%)	
Immunomodulators		17 (22.4%)
Azathioprine	2 (5.7%)	
Mercaptopurine	1 (2.9%)	
Corticosteroids		14 (18.4%)
Budesonide	3 (8.6%)	
Prednisone	5 (14.3%)	
Oral-aminosalicylates		
Sulfasalazine	2 (5.7%)	
Mesalamine	8 (22.9%)	
Anti-diarrheal		
Loperamide	1 (2.9%)	
Other		11 (14.5%)^e^
Missing		1 (1.3%)
Comorbid Conditions^a^, n (%)		
No other conditions	13 (37.1%)	29 (38.2%)
Allergic rhinitis	3 (8.6%)	7 (9.2%)
Anemia	2 (5.7%)	
Ankylosing spondylitis		1 (1.3%)
Anxiety	4 (11.4%)	5 (6.6%)^f^
Arthritis		
Enteropathic arthritis	1 (2.9%)	
Osteoarthritis	3 (8.6%)	1 (1.3%)
Rheumatoid arthritis	1 (2.9%)	6 (7.9%)
Asthma	3 (8.6%)	8 (10.5%)
Cancer	1 (2.9%)^g^	
Celiac disease		3 (3.9%)
COPD	1 (2.9%)	1 (1.3%)
Depression	4 (11.4%)	19 (25.0%)
Diabetes (Type 2)		2 (2.6%)
GERD	7 (20.0%)	
Hypertension	8 (22.9%)	12 (15.8%)
Multiple sclerosis	1 (2.9%)	1 (1.3%)
Psoriasis	1 (2.9%)	5 (6.6%)
Other autoimmune condition(s)		4 (5.3%)^h^
Other mental health condition(s)^		1 (1.3%)^i^
Other health conditions^a^	9 (26.0%)j	11 (14.5%)^k^
Missing		2 (2.6%)

*CDEIS* Crohn’s Disease Endoscopic Index of Severity, *COPD* chronic obstructive pulmonary disease, *CRP* C-reactive protein, *GAD* generalized anxiety disorder, *GERD* gastroesophageal reflux disease, *IIH* idiopathic intracranial hypertension, *Hep B* Hepatitis B, *MS* multiple sclerosis, *POTS* postural orthostatic tachycardia syndrome, *PTSD* post-traumatic stress disorder, *SD* standard deviation

^a^Responses are not mutually exclusive

^b^Specified employment responses (n = 1 each): “self-employed,” “unemployed – but have filed disability and am still battling in court at this time.”

^c^Other employment status specified by n = 1 each: “Short-term disability,” “Try to work and do some volunteering.”

^d^Other marital status specified by n = 1 each: “Divorced 19 years not married,” “Engaged.”

^e^Other treatment specified by n = 1 each except where specified otherwise: Mesalamine (n = 3), Medical marijuana/cannabis (n = 2), “A study medication,” Amitiza, Amitriptyline, Lialda, Pantoprazole, “Tacro.”

^f^Anxiety was not included as a response option in the questionnaire but as n = 5 participants wrote this in as an “other” response it has been specified in the table

^g^History of squamous cell on right calf

^h^Other autoimmune conditions specified by n = 1 each except where specified otherwise: Primary Sclerosing Cholangitis (n = 2), Hidradenitis suppurativa, “Liver issues,” Prurigo nodularis

^i^Other mental health conditions specified by the same n = 1 participant: PTSD and GAD

^j^Other health conditions reported in n = 1 participant each (not mutually exclusive): acid reflux, kidney stones, osteopenia, gastroesophagitis, irritable bowel syndrome (documented as well-controlled), neuropathy, fibromyalgia, obstructive sleep apnea, colon polyps, osteoporosis, chronic pain syndrome, “sleep disorder.”

^k^Other health conditions specified by n = 1 each except where specified otherwise: Fibromyalgia (n = 2), High cholesterol (n = 2), Ankylosis arthritis and scoliosis, fatty liver disease, GERD, Hep B (inactive now) and sleep apnea, hypothyroidism, IIH and POTS, leg lymphedema, Raynaud’s Syndrome

#### Test–retest reliability of the Urgency NRS

The stability and reproducibility of the Urgency NRS weekly score was assessed within a stable population by comparing the Week 1 and Week 2 assessments. Statistical significance was based on paired t-tests. Stable subjects were defined as having a response of “no change” on the PGIC on Day 14 as the primary method, followed all subjects who had a change less than |1| on their weekly scores between Weeks 1 and 2 in the PGRS, BM Count, and/or the abdominal pain NRS.

Interclass correlation coefficients (ICCs) and effect size (ES) for the Urgency NRS mean scores were calculated for Week 1 and Week 2. ICCs range from 0 to 1.0, with higher scores indicating a more stable instrument, and values > 0.70 generally indicating strong test–retest reliability [27, 28]. A minimal mean difference of < 0.20 in ES (i.e.; standardized mean difference) between Week 1 and Week 2, as well as lack of a significant difference (p > 0.05) were used to support stability of the Urgency NRS. Test–retest analyses were only conducted if there were ≥ 30 participants in the stable groups [29].

#### Construct validity of the Urgency NRS

Construct validity [27] of the Urgency NRS was assessed by comparing it with PROs of interest. Correlations were classified as small (< 0.3), moderate, (0.3 to 0.6), or large (> 0.6) [30]. A correlation coefficient greater than 0.3 indicated convergent validity [31]. A priori hypotheses specified expected moderate to large correlations between the Urgency NRS and the PGRS, BM Count, and the abdominal pain NRS, but small to moderate correlations with the fatigue-related measures (PGIS-Fatigue and FACIT-Fatigue).

#### Known-groups validity of the Urgency NRS

Known-groups validity is the extent to which scores from an instrument are distinguishable between groups of subjects that differ by a relevant clinical indicator [32]. To evaluate known-groups validity, the Week 1 score for Urgency NRS scale was analyzed by Week 1 Overall CD Symptom PGRS, Abdominal Pain NRS, and BM Count using the following groups:

- Overall CD PGRS: 0– < 3, 3–5
- Abdominal Pain NRS: Below and ≥ median score; 0–4 vs.5–10
- BM Count: Below and ≥ median score

The predefined groups were collapsed as necessary to ensure all comparison groups contained at least 10 participants. The analysis of variance (ANOVA) models included the Urgency NRS scores as the dependent variable and the known-group criterion variable as the independent variable to assess the significance of Week 1 mean differences for each group. Analyses were repeated with Week 2 scores.

## Results

### A. Qualitative interviews

#### Patient demographics and clinical characteristics

Thirty-five participants with a mean age of 45.1 years (SD 15.83) were recruited from 6 clinical sites. Participants were mostly female (65.7%), non-Hispanic or Latino (97.1%), and White (80.0%). Thirteen participants (37.1%) had a college degree whereas 11 (31.4%) had only finished high school/secondary school (Table 1).

Overall, the mean time since CD diagnosis was 11.9 years (SD 13.7). The most commonly reported treatments and comorbid health conditions are summarized in Table 1.

The relevance of BU as a symptom of CD is underscored by the observation that, of the 35 participants interviewed, 34 (97%) participants reported, either spontaneously (n = 13) or through probing (n = 21), experiencing BU that they attributed to their CD. BU was reported as one of the most bothersome CD symptoms by 15 participants, followed by increased abdominal pain/cramping (n = 12), frequency of BMs (n = 6), diarrhea (n = 5), and fatigue (n = 4). The highest proportion of participants indicating that BU was their most bothersome symptom had Type 4 [colonic involvement, with or without small bowel involvement (rectal+distal colon only)] (Supplemental Fig. 2).

**Fig. 2:**
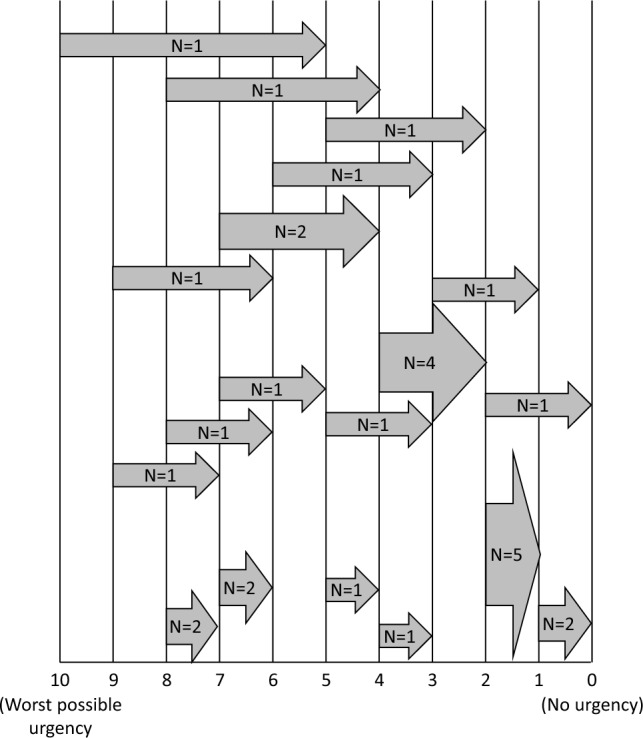
Meaningful Improvement on the Urgency NRS (N = 30). Participants provided their current Urgency NRS score, and the point where they felt a decrease would be meaningful. The flat end of the arrow represents the participants’ current scores, and the tip of the arrow represents the point at which the change would become meaningful to them. The numbers in the figure indicate the number of participants with that response across the full sample

#### Experience and frequency of BU

Table 2 and Supplemental Table 2 provide examples of quotes participants used to describe BU. Of the 34 participants who experienced BU due to CD, 22 (65%) reported that they experienced BU every day or with nearly every BM. Of the participants reporting BU every day, there was a trend suggesting that the highest proportions were among Types 1 or 2, and the least among Type 3. Half of the participants reported that BU fluctuated over time while the others reported it as stable or consistent.

**Table 2:** Common terminology used for “Urgency”

Terminology used	Participants n = 34 (%)	Anonymized participant ID, age, CD subtype, Supportive quote
“Got to go right now” or “When I have to go, I have to go”	8 (24)	200–010, 65, Type 3: *“Just when you feel like you have to go, you don’t put, don’t put it off”*
“Urge (urgency) to go to the bathroom”	6 (18)	700–006, 46, Type 2: *“I’d say just urge – urge to go to the bathroom after eating”*
“Immediacy”	5 (15)	700–001, 44, Type 1: *“Immediate need to use the bathroom”*
“Unable to hold it”	4 (12)	200–006, 29, Type 3: *“it just feels like, uh, just unable to hold it, um, again a lot of burning, pressure…It just kind of hits you out of nowhere. It's not really something you can plan for”*

*CD* Crohn’s Disease, *ID* anonymized participant identifier, *NRS* numeric rating scale

Most participants (n = 28) noted that BU is worse depending on certain foods or drinks. Eight participants indicated BU is worse in the morning, and three noted their BU can be triggered by certain activities (e.g., walking, exercising).

#### Severity of BU

BU severity was described in terms of time to get to toilet (e.g., having 2 to 3 min (n = 6), or ≤ 1 min (n = 4)), while others used descriptors such as “urgent” (n = 5), “severe” (n = 2), “desperate” (n = 1), “not very urgent” with time to get to toilet (n = 5). Still others rated the severity of pain (“six out of 10” (n = 1), or “painful” (n = 1).

#### Association of BU and frequency of BMs

Thirty-three participants were asked whether their BU and BM frequency were related, and 15 (45%) reported that BU and BM frequency always or usually co-occur (i.e., they tend to have more BU on days when they also have more frequent BMs), whereas 18 (55%) noted they could experience one symptom without the other.

#### Impacts of BU

Interview participants reported major impacts on their daily activities due to BU and resulting incontinence (Supplemental Table 3). The most commonly reported impacts involved recreation or hobbies, needing to always be aware of toilets when in public and having to stay home more. Twelve participants indicated a mental and/or emotional impact. Nine participants described a general impact on daily activities, noting that their BU prevents them from doing things that “normal” people can do. Participants spoke about having less anxiety around finding restrooms and avoiding accidents if their BU improved. Participants noted that they would not be able to leave the home if their BU was more severe.

**Table 3:** Participant descriptions of each level of urgency on the NRS

Bowel Urgency NRS severity range	Anonymized participant ID, age, CD subtype, Supportive quote
Mild	200–007, 36, Type 1: *“That I have no, no eminent like I need to go to the bathroom”*
Moderate	300–006, 34, Type 3: *“That would be I would say the sudden need to use the restroom but not like you feel like you’re going to have an accident”*
Severe	200–010, 65, Type 3: *“*[Severe] *would be like where you'd say within two or three feet* [from a toilet]*…I've had that before…you go and then you think you're done and then you walk back in and you turn around and walk back…It's horrible. It would be huge impact, you know, where you, you can't, you can't leave your house”*
Urgency when in CD Remission	200–007, 36, Type 1: *“Very minimal to no urgency. Being able to know I have to go and having that couple minutes to get myself there and not have to drop what I'm doing and rush”*

#### Responses to the Urgency NRS

The mean score for the cohort was 4.4 (SD 3.03), with a median score of 4.0 (Supplemental Table 4). The mean scores for the individual CD subtypes ranged from 3.2 (SD 2.99; Type 1 CD) to 5.4 (SD 3.00; Type 2 CD). Each of the response options were selected at least once.

**Table 4:** Urgency NRS Test–retest reliability: week 1 and week 2

Urgency NRS Scores^a^	N	Week 1 Mean (SD)	Week 2 Mean (SD)	Difference Score^b^	t-value	p-value^c^	Effect Size	ICC
Based on No Change in Overall CD Symptom PGIC (N = 37)	37	5.34 (2.30)	4.94 (2.41)	-0.40	− 2.26	0.0301	0.18	0.88
Based on No Change in Overall CD Symptom PGRS (N = 66)	66	4.95 (2.16)	4.49 (2.40)	-0.46	− 3.01	0.0037	0.21	0.84
Based on Bowel Movement Count (N = 52)	52	4.52 (2.13)	3.95 (2.28)	-0.57	− 3.63	0.0007	0.27	0.84
Based on Abdominal Pain NRS (N = 47)	47	4.66 (2.40)	4.25 (2.53)	-0.41	− 2.76	0.0082	0.17	0.90

*CD* Crohn’s disease, *ICC* intraclass correlation coefficient, *NRS* numeric rating scale, *PGIC* Patient Global Impression of Change, *PGRS* Patient Global Rating of Severity, *SD* standard deviation

^a^Among patients defined as having experienced no change in Overall CD symptoms as measured by the Overall CD Symptom PGIC total score, or in Overall CD Symptom PGRS, or Bowel Movement Count, or Abdominal Pain NRS at Day 14, as indicated

^b^Calculated as Week 2 Mean Score minus Week 1 Mean Score

^c^Paired t-tests comparing responses at Week 1 and Week 2

#### Cognitive debriefing of the Urgency NRS

##### Understanding and interpretation

All but one participant (3%) found the Urgency NRS item clear in meaning and easy to answer. The remaining participant reported having some trouble reading in general. Participants were able to differentiate the different severity levels of BU when asked to discuss why they selected a particular response on the Urgency NRS. Twenty-nine participants (83%) used the correct recall period (24 h) when responding, whereas 5 participants (14%) seemed to think only of the waking hours on the day of the interview; and 1 participant (3%) did not directly comment on their recall period.

##### Interpretation of mild, moderate, and severe urgency on the NRS

In general, participants assigned an Urgency NRS score of > 0–3 with “Mild”, > 3–7 with “Moderate”, and > 7–10 with “Severe” urgency (Fig. 1). Individual definitions of “Moderate” often overlapped with those for “Mild” and “Severe.” (Fig. 1). In general, BU while in CD remission correlated with the “mild” range, although some (n = 7) suggested they could have an Urgency NRS score as high as a 4 while still in CD remission’

Participants described BU at each severity level, as illustrated by selective quotes (Table 3; Supplemental Table 5). In general, the “Mild” range of BU was described as not requiring them to get to the restroom as quickly. “Moderate” BU was described as starting to be disruptive, but still manageable, as they had to get to the restroom more quickly. “Severe” BU was equated with not being able to leave the home and having to stay very close to a toilet at all times, significantly impacting daily activities and HRQOL. Thirty-three participants were asked what their BU would be like if they were in CD remission; 15 participants (45% of those answering the question) said that they would have none, whereas 17 participants (52%) said they could still experience some (2–4 on the Urgency NRS) BU.

**Table 5:** Urgency NRS construct validity correlations with overall CD symptom PGRS, bowel frequency count, abdominal pain NRS, PGIS-Fatigue and FACIT Fatigue

Measures	Correlation	n	p-value
Week 1 (N = 76)			
Overall CD Symptom PGRS (Week 1)	0.71	76	< .0001
Bowel Movement Count (Week 1)	0.53	75	< .0001
Abdominal Pain NRS (Week 1)	0.65	76	< .0001
PGIS-Fatigue (Day 7)	0.44	64	0.0002
FACIT-Fatigue (Day 7)	− 0.45	64	0.0002
Week 2 (N = 74)			
Overall CD Symptom PGRS (Week 2)	0.77	74	< .0001
Bowel Movement Count (Week 2)	0.50	73	< .0001
Abdominal Pain NRS (Week 2)	0.73	74	< .0001
PGIS-Fatigue (Day 14)	0.48	66	< .0001
FACIT-Fatigue (Day 14)	− 0.53	66	< .0001

*CD* Crohn’s disease, *FACIT-Fatigue* Functional Assessment of Chronic Illness – Fatigue, *NRS* numeric rating scale, *PGIS* Patient Global Impression of Severity, *PGRS* Patient Global Rating of Severity

##### Interpretation of PGRS

Twenty-seven participants (77%) reported that BU was a factor in their response; 12 mentioned this spontaneously, and 15 confirmed when probed. *Interpretation of PGIC*. When asked to describe their thoughts while deciding how to answer the Overall CD Symptom PGIC, 7 participants (20%) reported thinking about abdominal pain or cramping, 4 (11%) about the number or frequency of bowel movements, and 4 (11%) about BU. Ten participants (29%) spoke more generally about considering different medications they had been on and comparing how they felt at various points to the present time.

#### Meaningful change on the Urgency NRS

Using their current score as a starting point, 13 participants (starting Urgency NRS score range: 1–8) reported that a reduction of 1 point would be a meaningful improvement, and another ten participants (starting Urgency NRS score range: 2–9) indicated that a reduction of 2 points would be meaningful (Supplemental Table 6; Fig. 2). All 7 participants who indicated that a minimum improvement of 3, 4, or 5 points would be needed had a starting score of ≥ 5 (Supplemental Fig. 2).

**Table 6:** Known-groups validity of the Urgency NRS

Anchor	Urgency NRS Average week 1 score		Overall F-test		Effect size^a^
	N	Mean (SD)	p-Value		
Week 1 (N = 76)					
Overall CD PGRS			< .0001		1.305
0– < 3	38	3.48 (1.98)			
3–5	38	5.95 (1.80)			
Bowel Movement Count			0.0002		0.897
< Median	37	3.73 (2.08)			
≥ Median	38	5.55 (1.94)			
Abdominal Pain			< .0001		1.201
< Median	38	3.55 (1.99)			
≥ Median	38	5.88 (1.89)			
Abdominal Pain			< .0001		1.770
0–4	51	3.79 (1.96)			
5–10	25	6.60 (1.56)			
Week 2 (N = 74)					
Overall CD PGRS			< .0001		1.933
0– < 3	42	2.85 (1.63)			
3–5	32	6.12 (2.01)			
Bowel Movement Count			0.0003		0.899
< Median	37	3.24 (2.05)			
≥ Median	36	5.16 (2.25)			
Abdominal Pain			< .0001		1.489
< Median	36	2.76 (1.77)			
≥ Median	38	5.69 (2.08)			
Abdominal Pain			< .0001		2.525
0–4	52	3.17 (1.77)			
5–10	22	6.86 (1.67)			

*CD* Crohn’s disease, *FACIT-Fatigue* Functional Assessment of Chronic Illness – Fatigue, *ICC* intraclass correlation coefficient, *NRS* numeric rating scale, *PGRS* Patient Global Rating of Severity, *SD* standard deviation

^a^Calculated by absolute delta mean score dividing by the pooled standard deviation

### B. Quantitative survey

#### Participant demographics and clinical characteristics

Of the 76 participants who completed the web-survey, 16 (21.1%) had also completed the interview as part of the earlier study component, whereas 60 were newly recruited. The mean age of the sample was 41.9 years (SD 13.24). Participants were mostly female (n = 50; 65.8%), White (n = 63; 82.9%), non-Latino or Hispanic (n = 72; 94.7%) and had a college or postgraduate degree (n = 60; 78.9%) (Table 2). Disease characteristics are summarized in Table 2.

#### PRO descriptive characteristics for Urgency NRS

A total of 76 participants completed the Baseline/Day 1 survey entry, 64 participants (84.2%) completed Day 7, and 66 participants (86.8%) completed Day 14. There were sufficient responses to calculate mean scores for 76 participants (100%) for Week 1 and 74 participants (97.4%) for Week 2. One participant opted out of the BM Count item during each of their survey entry days, but there were no other missing responses across any of the other PRO instruments.

Mean scores for the Urgency NRS were 4.7 (SD 2.26) at Week 1 and 4.3 (SD 2.42) at Week 2 (Table 4). The medians (range) were 4.5 (0–10) and 4.0 (0–10) for Weeks 1 and 2, respectively. Floor and ceiling effects were minimal, with 1 (1.3%) participant having minimal or maximal values at Week 1, and 1 (1.4%) participant having minimal and 2 (2.7%) having maximal values at Week 2. The frequency distribution of NRS scores is shown in Supplemental Fig. 3.

#### Test–retest reliability

Thirty-seven participants responded “No Change” to the PGIC at Day 14. The ICC was 0.88, indicating strong test–retest reliability. However, the effect size of 0.18 was just below the maximum acceptable level for supporting stability of the score and the Urgency NRS score difference between the assessments at Week 1 and 2 was statistically significant (p = 0.03) (Table 4). Test–retest reliability within stable population using the PGRS, BM Count or Abdominal Pain NRS showed strong reliability based on the ICC (Table 4).

#### Construct validity for the Urgency NRS

At Week 1, the Urgency NRS score was highly correlated with the PGRS and the Abdominal Pain NRS (0.71 and 0.65, respectively), and it was moderately correlated (0.44 to 0.53) with the remaining PRO measures (Table 5). All correlations were statistically significant and within the a priori hypotheses. At Week 2, the Urgency NRS score was more highly correlated with the PGRS and the Abdominal Pain NRS (0.77 and 0.73, respectively). These correlations were higher than hypothesized a priori. All other correlations were moderate (|0.48| to |0.53|) and were again within the hypothesized ranges (Table 5).

#### Known-groups validity of the Urgency NRS

Each of the predefined severity groups contained at least 10 participants and no further group collapsing was necessary. As expected, more severe Urgency NRS scores were associated with more severe responses to the selected PROs (PGRS, BM Count, and Abdominal Pain NRS). All comparisons were statistically significant at both Weeks 1 and 2 (Table 6).

## Discussion

BU is an important, relevant symptom of CD that varies in intensity and is highly valued in treatment goals to improve HRQOL [8, 13]. As such, daily assessment of BU severity should be considered as a clinical endpoint when designing clinical trials. The interviews with respondents documented the burdensome nature of BU on the patient, and that it greatly impacts HRQOL. Here, the proportions of participants experiencing BU are higher than those reported in some other reports [7]. Clinical and physical assessments of IBD do not provide an accurate measure of the patient’s own perception of their disease and their HRQOL [33]. This dissociation between a patient’s perception of their HRQOL and clinical measurements of disease activity has been demonstrated with patients who have other diseases [33], underlining the importance of devising reliable, reproducible measurements that can help assess the severity of patients’ symptoms from their perspective.

This rating scale has been validated in patients with UC [18, 34]. The results presented here support its use in CD. Findings indicate that the Urgency NRS is a simple, reliable, reproducible, valid, and interpretable PRO scale that can be used to assess one of the most troublesome CD symptoms. We found strong support for content validity for the Urgency NRS in both the qualitative interviews and the web survey, and a large majority of participants endorsed BU as a symptom of their CD in qualitative interviews. Participants could define different levels of BU severity, describe daily life impacts, and score them differently on the Urgency NRS. Patients largely agreed on the rating range for mild urgency (scores of 1–3 on the Urgency NRS), described as “normal urgency”, moderate urgency (scores of 4–7 on the Urgency NRS) and severe urgency (scores > 7 on the Urgency NRS). These thresholds could aid patients, healthcare providers, and other stakeholders in interpreting the Urgency NRS score, although further studies are needed to quantitatively assess within patient responder thresholds.. In addition, it will be important to further explore patient perceptions regarding symptom remission, given that our exploratory results indicate that some patients feel that CD remission is a total absence of symptoms, whereas others indicated that mild symptoms could be present in remission.

The web survey results also provide strong support of content validity for the Urgency NRS, with very minimal floor effects at both Week 1 and Week 2. The findings from the web survey also indicate that the Urgency NRS has good measurement properties. Test–retest reliability of the Urgency NRS was strong when using the PGRS and BM Count as anchors, and moderate when using the PGIC. Construct and known-groups validity against PGRS, BM Count, the Abdominal Pain NRS, PGIS-Fatigue, and FACIT-Fatigue scores were overall strong and within ranges hypothesized a priori. This further supports previous studies that found that the coexistence of BU, fatigue, and abdominal pain are especially burdensome for CD patients [35].

## Limitations

Recruitment for the qualitative interviews was limited by available patient pools and recruitment timelines, and so the sample might not be representative of the greater CD population. This is a limitation commonly cited for qualitative research, especially in those with smaller sample sizes [36]. The purpose of the qualitative research was to assure content validity rather than to make generalizable conclusions.

In examining test–retest reliability, score ranges were used instead of the response categories to assess known groups for the overall symptom PGRS because it used a 24-h recall period and an average score was calculated over 7 days. The analysis plan used smaller ranges of numbers corresponding to the discreet verbal categorical responses (0– < 1 = “None,” 1– < 2 = “Very Mild,” etc.). However, final groupings were collapsed due to insufficient N in some of the categories (i.e., a priori, we had stated that group sizes < 10 would be collapsed).

The web survey was a pilot study to assess the psychometric properties, but the findings should be confirmed with clinical trial data. The majority of web survey participants were recruited via a research recruitment vendor and thus may not be representative of other CD patients in the US. Although CD diagnosis was confirmed for each of these participants, no clinical data was available to clinically define or confirm disease severity. Many participants were unaware of their CD subtype, and we were unable to verify responses for those who did report a subtype. Another limitation was that a healthy control group was not included.

## Conclusions

BU is an important symptom of CD. The evidence provided herein demonstrates that the Urgency NRS has content validity, test–retest reliability, and construct validity. The Urgency NRS is a well-defined and reliable PRO instrument that is suitable to be used in clinical studies to evaluate a treatment benefit in patients with CD.

### Supplementary Information

Below is the link to the electronic supplementary material.

Supplementary file1 (DOCX 675 KB)

## Data Availability

Not applicable.
