# Supplementary material for: Validation of the bowel urgency numeric rating scale in patients with Crohn’s disease: results from a mixed methods study
Source: Qual Life Res. 2023 Aug 4;32(12):3403–15. doi: 10.1007/s11136-023-03494-y (PMC10624712; doi:10.1007/s11136-023-03494-y)
Supplement: Supplementary file 1 — Supplementary file1 (DOCX 675 KB) [file 11136_2023_3494_MOESM1_ESM.docx]

| Supplemental Table 1. Survey Administration Schedule | | | | | |
| --- | --- | --- | --- | --- | --- |
| Patient-reported Outcome | Number of Items | Baseline/ Day 1 | Days 2 through 6, 8 though 13 | Day 7 | Day 14 |
| Sociodemographic and Clinical Characteristic Questionnaire | 11 | ✓ |  |  |  |
| Overall CD Symptom PGRS | 1 | ✓ | ✓ | ✓ | ✓ |
| Overall CD Symptom PGIC | 1 |  |  |  | ✓ |
| Abdominal Pain NRS | 1 | ✓ | ✓ | ✓ | ✓ |
| BM Count | 1 | ✓ | ✓ | ✓ | ✓ |
| Urgency NRS | 1 | ✓ | ✓ | ✓ | ✓ |
| FACIT-Fatigue | 13 | ✓ |  | ✓ | ✓ |
| PGIS-Fatigue | 1 | ✓ |  | ✓ | ✓ |
| PGIC-Fatigue | 1 |  |  |  | ✓ |
| RAND-36 | 36 | ✓ |  |  |  |
| Total items administered at each time point | N/A | 65 | 4 | 18 | 20 |
| Abbreviations: CD = Crohn’s disease; FACIT-Fatigue = Functional Assessment of Chronic Illness Therapy – Fatigue; NRS = numeric rating scale; PGIC = Patient Global Impression of Change; PGIS = Patient Global Impression of Severity; PGRS = Patient Global Rating of Severity | | | | | |

| Supplemental Table 2. Common Terminology Used for “Urgency” | | |
| --- | --- | --- |
| Terminology  used | Participants n=34 (%) | Anonymized participant ID, age, CD subtype, Supportive quotes |
| “Got to go right now” or “When I have to go, I have to go” | 8 (24%) | 600-005, 49, Type 3: *“Yeah, you got to go, you got to go, yeah, that's which I normally say.”*  200-001, 61, Type 4: *“You got to go right now. Yeah. There's no waiting around.”*  200-010, 65, Type 3: *“Just when you feel like you have to go, you don’t put, don’t put it off.”*  600-007, 20, Type 1: *“When I got to go, I got to go.”*  100-005, 54, Type 2: *“…it would be here like it says here* [on the Urgency NRS questionnaire], *sudden or immediate need. It—yeah. It's just, you get—you just got, you just got to go.”* |
| “Urge (urgency) to go to the bathroom” | 6 (18%) | 700-006, 46, Type 2: *“I’d say just urge – urge to go to the bathroom after eating.”*  600-003, 28, Type 5: *“Urgency is really the only way I've ever described it. I don’t know if, I don’t know if I know another way to describe it.”*  300-004, 41, Type 1: *“urgency is probably the better term, I can’t think of how I would say it. I can’t think of how I could say it without using that word.”* |
| “Immediacy” | 5 (15%) | 700-001, 44, Type 1: *“Immediate need to use the bathroom.”*  300-003, 39, Type 2: *“They would just look at me and could tell I need something immediately to a restroom or something immediately.”*  100-002, 69, Type 2: *“Urgency, immediacy.”*  300-003, 39, Type 2: *“They would just look at me and could tell I need something immediately to a restroom or something immediately.”* |
| “Unable to hold it” | 4 (12%) | 200-006, 29, Type 3: *“it just feels like, uh, just unable to hold it, um, again a lot of burning, pressure…It just kind of hits you out of nowhere. It's not really something you can plan for.”*  100-003, 23, Type 5: “*I would just say it's like you can't hold it. You have to go…it's…almost like vomiting. You just, you just have to do it. There's no break. There's no like you can think about it. You just have to use the bathroom.”*  600-004, 66, Type 2: “*Oh, I just like, uh, I just try to hold—I try to hold those muscles I guess together so that I, so that I can wait. And sometimes it's like I can hardly walk to get to the toilet and sit down before I let loose.”* |
| Supportive quotes include the anonymized participant ID, age, type of CD, and quote.  The CD subtypes are:  Type 1: Small bowel involvement only, including isolated ileitis;  Type 2: Colonic involvement, with or without small bowel involvement (Proximal ± Transverse Colon only);  Type 3: Colonic involvement, with or without small bowel involvement (rectal only);  Type 4: Colonic involvement, with or without small bowel involvement (rectal + distal colon only); and  Type 5: Colonic involvement, with or without small bowel involvement (pancolitis). | | |

| Supplemental Table 3. Impacts of Urgency Reported by at Least 2 Participants | |
| --- | --- |
| Impacts of Urgency | Total  N=34 (97%) ^a^ |
|  | **Reported n (%)^b^** |
| Recreation or hobbies | 22 (65%) |
| Needing to be aware of toilets | 20 (59%) |
| Staying home more | 19 (56%) |
| Anxiety or depression (caused by urgency) | 14 (41%) |
| Social activities | 14 (41%) |
| Nutritional or diet impact | 14 (41%) |
| Work | 13 (38%) |
| Family life | 12 (35%) |
| General mental or emotional impact (not specified further) | 12 (35%) |
| General daily activities or life impact | 9 (26%) |
| Frustration, anger, or annoyance | 9 (26%) |
| Embarrassment | 7 (21%) |
| Travel or driving | 6 (18%) |
| Sleep | 5 (15%) |
| Needing to carry extra clothing | 3 (9%) |
| Draining, causes fatigue | 3 (9%) |
| Losing time or missing events | 3 (9%) |
| Yard or housework | 3 (9%) |
| ^a^  Number and proportion of the full sample who reported urgency; one participant is missing  ^b^ Number and proportion reporting the impact within those reporting urgency, within the full sample | |

| Supplemental Table 4. Participant Responses to the Urgency NRS By CD Subtype | | | | | | |
| --- | --- | --- | --- | --- | --- | --- |
| Urgency  NRS Response | Type 1 n=6 (17%) | Type 2 n=9 (26%) | Type 3  n=7 (20%) | Type 4 n=5 (14%) | Type 5 n=8 (23%) | Total  N=35 (100%) |
| Mean score (SD) | 3.2 (2.99) | 5.4 (3.00) | 3.4 (3.36) | 4.8 (2.59) | 4.8 (3.24) | 4.4 (3.03) |
| Median score [Range] | 3.0 [0-7] | 5.0 [2-10] | 2.0 [0-8] | 4.0 [2-9] | 5.5 [0-9] | 4.0 [0-10] |
| Participants selecting each individual response, n (%) | | | | | | |
| 0 | 2 (33%) | 0 (0%) | 1 (14%) | 0 (0%) | 1 (13%) | 4 (11%) |
| 1 | 0 (0%) | 0 (0%) | 2 (29%) | 0 (0%) | 1 (13%) | 3 (9%) |
| 2 | 1 (17%) | 3 (33%) | 1 (14%) | 1 (20%) | 0 (0%) | 6 (17%) |
| 3 | 0 (0%) | 0 (0%) | 0 (0%) | 0 (0%) | 1 (13%) | 1 (3%) |
| 4 | 1 (17%) | 0 (0%) | 1 (14%) | 2 (40%) | 1 (13%) | 5 (14%) |
| 5 | 0 (0%) | 2 (22%) | 0 (0%) | 1 (20%) | 0 (0%) | 3 (9%) |
| 6 | 1 (17%) | 0 (0%) | 0 (0%) | 0 (0%) | 0 (0%) | 1 (3%) |
| 7 | 1 (17%) | 1 (11%) | 0 (0%) | 0 (0%) | 3 (38%) | 5 (14%) |
| 8 | 0 (0%) | 2 (22%) | 2 (29%) | 0 (0%) | 0 (0%) | 4 (11%) |
| 9 | 0 (0%) | 0 (0%) | 0 (0%) | 1 (20%) | 1 (13%) | 2 6%) |
| 10 | 0 (0%) | 1 (11%) | 0 (0%) | 0 (0%) | 0 (0%) | 1 (3%) |
| Abbreviations: n=numbers of participants; NRS = numeric rating scale.  The CD subtypes are:  Type 1: Small bowel involvement only, including isolated ileitis;  Type 2: Colonic involvement, with or without small bowel involvement (Proximal ± Transverse Colon only);  Type 3: Colonic involvement, with or without small bowel involvement (rectal only);  Type 4: Colonic involvement, with or without small bowel involvement (rectal + distal colon only); and  Type 5: Colonic involvement, with or without small bowel involvement (pancolitis). | | | | | | |

| Supplemental Table 5. Participant Descriptions of Each Level of Urgency on the NRS | |
| --- | --- |
| Bowel Urgency NRS Severity Range | Anonymized participant ID, age, CD subtype, Supportive quotes |
| Mild | 200-007, 36, Type 1: *“That I have no, no eminent like I need to go to the bathroom.”*  600-004, 66, Type 2: *“I know a bowel movement is coming on, but you don’t have to move right now to get there.”*  700-004, 50, Type 3 *“you have to go but you're not beating down the door to go.”*  300-007, 50, Type 5: *“It would be almost like, um -- almost like, uh, any normal person who had to like go to the bathroom.”* |
| Moderate | 300-006, 34, Type 3: *“That would be I would say the sudden need to use the restroom but not like you feel like you’re going to have an accident.”*  200-002, 33, Type 5: *“Um, you know you've gotta get to the bathroom, um, you rush a little bit to do that, but you're not in a panic mode.”*  200-006, 29, Type 3: *“Moderate would be, um, you'd probably be having to go multiple times a day, so it's going to be interfering with your life a little bit…some of the things that are going to require a bathroom for a while, you're not going to want to do…or you're going to go there and probably not be enjoying yourself because you're going to be worrying the entire time about, you know, where a bathroom is...”*  100-002, 69, Type 2: *“That's like in the middle kind of, you know, it's not, not, not really severe but you've got to really be aware of what you're eating and what you're doing.”*  200-001, 61, Type 4: *“Well,* [moderate] *would be where you were a little more concerned but still able to wait patiently for other people to exit the bathroom. You're not learning. You're not dashing around like a crazy person with your eyes darting and watering.”* |
| Severe | 200-010, 65, Type 3: *“*[Severe] *would be like where you'd say within two or three feet* [from a toilet]*…I've had that before…you go and then you think you're done and then you walk back in and you turn around and walk back…It's horrible. It would be huge impact, you know, where you, you can't, you can't leave your house.”*  700-006, 46, Type 2: *“Well, I would have to stay at home. Couldn’t do nothing. I’d have to stay handcuffed to the toilet.”*  700-007, 46, Type 4: *“It would be, you know, a step up from the moderate where you definitely know you don't want to leave your house. You know, missing out on literally daily activities that you normally do, just not doing them whether it's…going to the gym, trying to run to the grocery store, you know, enjoy an hour-long baseball game, things like that you can't do because you're worried about the bathroom.”*  200-012, 56, Type 4: *“Like immediate, like almost immediate, like I have to have the bathroom now, I have no time to wait…it would affect my performance at work. Well, I didn’t make it to the restroom so I’ve got to leave work, and I’d have to tell somebody why, you know, and that's a little embarrassing, or if I’m out in public, somebody will see it or whatever.”*  300-003, 39, Type 2: *“I would have no seconds to wait.”* **INTERVIEWER: *“Okay. And how would that impact your daily life?”*** 300-003: *“It impacts it badly every day. It's just, like I said, I don't get to do things with my kids now at school events.”* |
| Urgency when in CD Remission | 200-007,36, Type 1: *“Very minimal to no urgency. Being able to know I have to go and having that couple minutes to get myself there and not have to drop what I'm doing and rush.”*  300-004, 41, Type 1: *“I would say it would be more like I wouldn't have any* [urgency]*, or if I did, I would be able to control it and wait for the right time instead of stopping my normal life to do it.”*  **INTERVIEWER: *“And what's the urgency like at that level?”*** 700-001, 44, Type 1*: “Like very little to none.”* **INTERVIEWER: *“Okay. And then would that impact your life if you were in remission?”*** 700-001: *“It affects you psychologically in certain ways. I think it might take a little while to get back to normal, but I think it's possible if you were in remission long enough that you could get back to normal activity.”*  300-003, 39, Type 2: *“*[In remission the urgency] *would be 1. I'd have it under control. I'd be okay maybe. I remember those days. I remember five years ago I was able to go to work every day, take my kids to events, to go watch my kid play football. I can't do those things now.”* |
| Abbreviations: CD = Crohn’s Disease; ID = anonymized participant identifier; NRS = numeric rating scale.  The CD subtypes are:  Type 1: Small bowel involvement only, including isolated ileitis;  Type 2: Colonic involvement, with or without small bowel involvement (Proximal ± Transverse Colon only);  Type 3: Colonic involvement, with or without small bowel involvement (rectal only);  Type 4: Colonic involvement, with or without small bowel involvement (rectal + distal colon only); and  Type 5: Colonic involvement, with or without small bowel involvement (pancolitis). | |

| Supplemental Table 6. Level of Improvement/Worsening on Urgency NRS that is Meaningful to Participants | |
| --- | --- |
|  | Total  N=35 (%) |
| Level of **improvement** that would be meaningful | |
| 1-point reduction | **13 (37%)** |
| 2-point reduction | **10 (29%)** |
| 3-point reduction | **5 (14%)** |
| ≥4-point reduction | **2 (6%)** |
| Not discussed | **5 (14%)** |
| Level of **worsening** that would be meaningful | |
| 1-point increase | **5 (14%)** |
| 2-point increase | **13 (37%)** |
| 3-point increase | **7 (20%)** |
| ≥4-point increase | **7 (20%)** |
| Not discussed | **3 (9%)** |
| Abbreviations: n=numbers of participants; NRS = numeric rating scale. | |

**Supplemental Figure Legends**

Supplemental Figure 1. PROs of interest. The Urgency NRS is a single-item questionnaire that asks respondents to rate the severity of their urgency with a recall period of 24 hours, using a 0 to 10 NRS (0 = “No urgency” and 10 = “Worst possible urgency”). The Patient Global Rating of Severity (PGRS) is a single-item PRO assessing the severity of CD symptoms overall in the past 24 hours using a six-level response scale of “None (0),” “Very Mild (1),” “Mild (2),” “Moderate (3),” “Severe (4),” and “Very Severe (5).” The Patient Global Impression of Change (PGIC) is a single-item questionnaire that assesses the change in CD symptoms overall since before starting to take a medication using a seven-level response scale ranging from “Very much better,” “Much better,” “A little better,” “No change,” “A little worse,” “Much worse,” and “Very much worse.”

Supplemental Figure 2. Urgency Endorsed and Most Bothersome. This figure shows the percentage of participants with each subtype of CD who endorsed BU as a symptom and the percentage of participants endorsing that symptom who considered it to be most bothersome, within the subtype and overall.

Supplemental Figure 3. Distribution of Urgency NRS Item Response Frequencies: Week 1 (A) and Week 2 (B). The Urgency NRS item was administered daily with a 24-hour recall period; the Week 1 item response frequencies are the percentages of participants with a mean score over days 1-7 (inclusive) within the listed range. Week 2 item response frequencies are the percentages of participants with a mean score over days 8-14 (inclusive) within the listed range.

**Supplemental Figure 1**

**
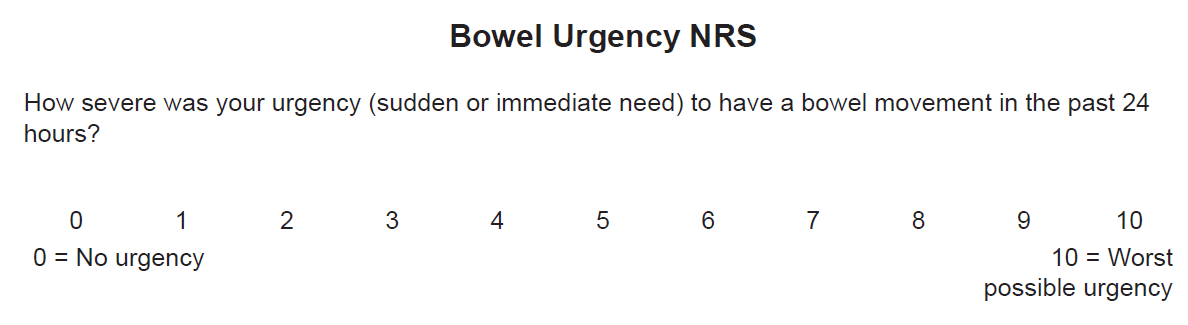
**

**
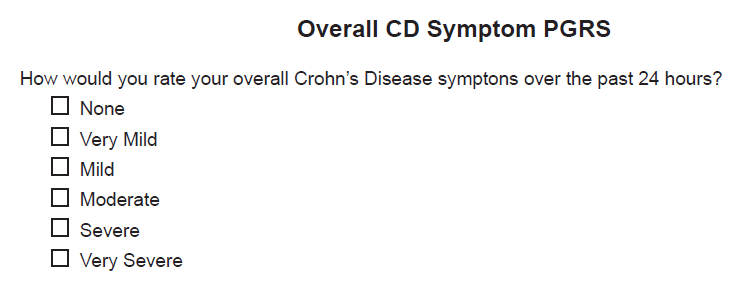
**

**
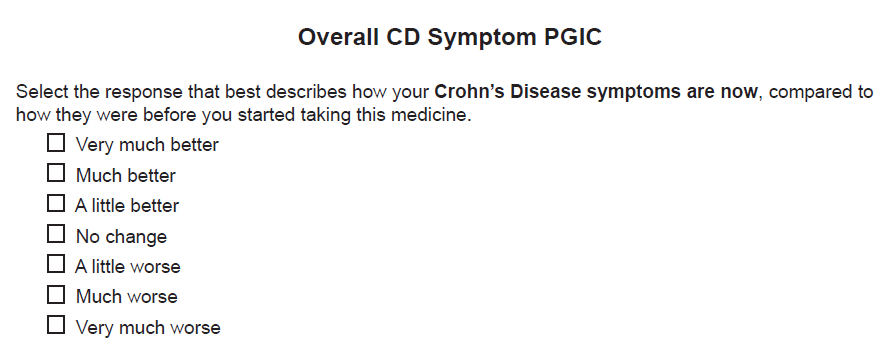
**

**Supplemental Figure 2**

**Supplemental Figure 3**
